# A Multifunctional Double-Array Petals Flower-Shaped Microfluidic Chip Combining Affinity and Physical Properties in Isolation of CTCs

**DOI:** 10.3390/mi17070811

**Published:** 2026-07-03

**Authors:** Hongmei Chen, Peng Zhang, Guosheng Peng, Houtong Liu

**Affiliations:** 1 School of Mathematics and Physics of Science and Engineering, Anhui University of Technology, Maanshan 243002, China; hongmeichen2015@sinano.ac.cn (H.C.);; 2 Physics Department, University of Massachusetts Lowell, Lowell, MA 01854, USA

**Keywords:** circulating tumor cells (CTCs), microfluidics, simulation

## Abstract

Circulating tumor cells (CTCs) are tumor cells that break away from the origin tumors and disseminate in the bloodstream and lymphatic circulation systems. CTCs originate from the original tumor with a similar bimolecular source. This makes CTCs play a vital status in cancer prognosis and diagnosis. However, CTC separation is highly challenging due to rarity and heterogeneity. In the present work, we designed a double-array petal flower-shaped microfluidic chip, a multifunctional capturing and isolation chip combining affinity and physical properties. The chip is composed of three arrays of microfluidic barriers organized one after the other. For the first array, six convex structures are set in each narrow channel. The first structure has a total of 12 such channels, which can increase collision frequency between cancer cells and convex structures in the channel. The second capture structure is one composed of an S-shaped array of concave triangle microcolumns and parabolic circular microcolumns. The advantage of this setting is that it can capture CTCs in the blood flowing into the first structure in 12 directions from multiple angles and multiple times, so as to improve capture efficiency. The third capture structure is composed of elliptical microposts and cylinders. The treated blood is captured for the last time. Because of the round or elliptical shape, it can retain the cell viability to a great extent, which is convenient for later pathological analysis of tumor cells. Simulation of velocity influence, pressure effects, streamline tendency, and shear rates is carried out for each structure. Therefore, theoretical validation has been illustrated to achieve high capture rate and purity. These delicate designs and numerical analysis clarify feasibility for further experiments of CTC enumeration, clinical analysis, and evaluation of cancer therapy.

## 1. Introduction

Circulating tumor cells (CTCs) refer to cancer cells that fall from the original location of cancer, enter the blood, and migrate to other places with the blood flow to increase in value [1,2]. The culprit of cancer metastasis we usually know is circulating tumor cells. For example, after cancer patients have undergone cancer cell resection surgery, it is likely that some tumor cells will enter the human blood to participate in the blood circulation. Once they find a place suitable for their own growth, they will grow at this new location; this is called cancer metastasis [3,4]. Cancer metastasis is the direct cause of death of most cancer patients [5,6]. Circulating tumor cells were first found by Thomas Ashworth in the blood of cancer patients, and were similar to the original cancer cells [7,8]. Therefore, it was speculated that cancer metastasis was closely related to these similar cells, but unfortunately, research was limited to the technology at that time and had no deep study of this phenomenon [9,10]. Finally, they were defined as circulating tumor cells by Pachmann in 2005 [11,12].

Microfluidic chips refer to devices that use micron level fluid channels to process fluid. Microfluidic chips are mainly based on in vitro detection [13,14], and the final analysis of in vitro detection is to detect and analyze DNA sequence [15,16] and spatial structure [17,18]. The analysis of tumor cells from the perspective of genes will be more accurate, which is of great significance for the targeted treatment of different cancers [19,20] and the exploration of the root causes of cancer [21,22,23]. Microfluidic chips are well suited to CTC isolation, based on their cell-sized capture sites, low chemical reagent consumption, and high throughput such as circular spiral chips. Roughly, CTC separation methods are based on two types: physical properties such as volume, density and electrical properties, and molecular biological characteristics such as infiltration ability and immunomagnetism. For affinity-based approaches, S. Nagrath et al. developed CTC-chip [24], a grapheme oxide microfluidic chip [22] for sensitively and successfully isolating CTCs from cancer patient blood. The herringbone chip greatly enhanced collision chances for CTCs and surfaces of microchannels [21]. For physical differences, Majid Ebrahimi Warkiani et al. presented a slanted spiral microfluidic chip for ultra-fast, label-free, and high efficiency isolation of CTCs. Sarioglu et al. developed a microfluidic device for sensitively isolating CTC clusters based on a label-free, physical capture approach [25,26,27,28,29]. In addition, there are microfluidic rachets [29] and a multi-obstacle architecture (MOA) filter [30] for isolation of CTCs based on physical properties. CTC isolation based on size and deformability differences [31,32,33,34,35,36] has the advantages of simplicity, easy fabrication, low cost, and high efficiency.

In this paper, a double-array petal flower-shaped microfluidic chip based on affinity and physical properties is designed. The aims and objectives are to design a unique microfluidic chip to realize high capture efficiency without yielding isolation purity. Theoretical validation could be carried out with software to prove the feasibility. This is a necessary step before experimental fabrication and clinical assays to optimize the chip according to theoretical study. AutoCAD2026 software is used to model the design drawings of the microfluidic chip, and COMSOL6.4 software is used to simulate various structures in the chip. Through simulation and analysis, the velocity field, pressure field, streamline tendency and shear effect, capture principle, and capture efficiency of the chip are deeply understood. The first structure uses the affinity principle to capture tumor cells for the first time. Due to the specificity of antigen–antibody binding, this structure can be used to achieve specific capture of tumor cells. According to the simulation results, it can also be seen that this structure has strong capture ability. The subsequent structures are based on the physical size required to capture tumor cells. Because of the petal structure, the number of microposts is large, which can realize multiple captures. In the later stage, it can be reasonably optimized according to the results shown in the simulation diagram. Based on the above results, the chip design is reasonable and feasible.

## 2. Materials and Methods

### 2.1. Simulation

An example is given in the following section about how to perform the COMSOL simulation.

Open the AutoCAD software and draw an ellipse centered at the origin with the major axis along Y-axis with length of 300 μm, and the minor axis along the X-axis with a length of 190 μm. Take the upper half of the ellipse, and draw seven circles with a diameter of 100 μm for each. Those circles are spaced at 8 μm apart, and with their centers all on the upper half of the ellipse. Draw a rectangle centered at the origin with a length of 900 μm and a width of 550 μm. Remove the elliptical portion and save the file as a dxf file.

**Figure 1:**
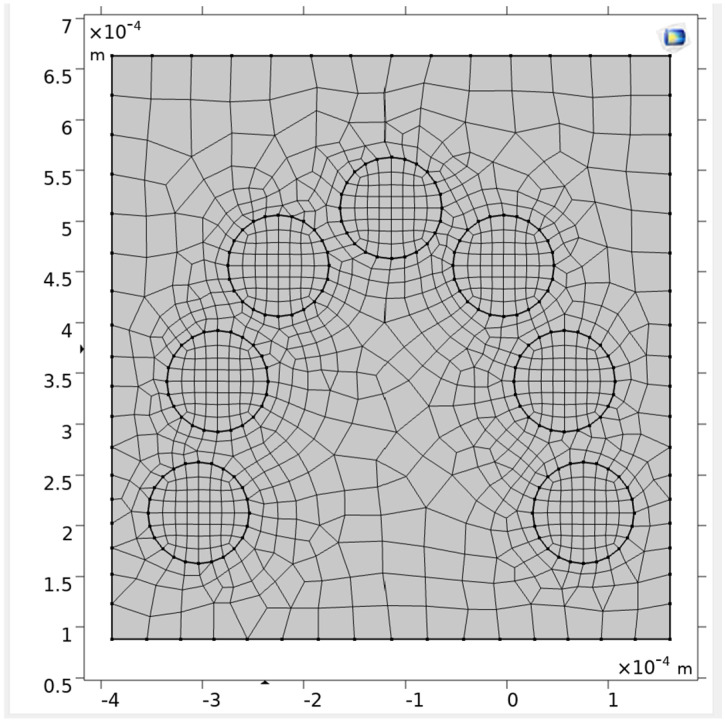
Computational mesh for simulation for the parabolic circular microposts array.

Open COMSOL software, click the Model Wizard, select 2D spatial dimensions, and in the physics field selection, choose Fluid Flow, Laminar Flow, and One-way Flow in sequence. Select Add and proceed to the Study Options, where you can choose the Steady-State option under General Studies. After setting the conditions, locate the Geometry button in the Model Builder, right-click, and import the previously drawn dxf file. Verify that the imported model is correct. In the Model Builder, right-click the Laminar Flow option and add an inlet and outlet. Set the inlet to the lower short edge of the rectangle with a velocity of 4 μm/s, and the outlet to the upper short edge with a pressure of 0 Pa. Right-click the Mesh option in the Model Builder, select Free Quadrilateral Mesh, and choose the entire geometry as the domain. Click Build Selected Objects to complete the meshing as shown in Figure 1. Finally, in the Model Builder, click the Study option, select Compute, and wait for the simulation results.

### 2.2. Isolation Values

Capture efficiency (%) = CTCs captured in the chip/total CTCs introduced (including RBCs, WBCs, and CTCs) × 100%

Or

Capture efficiency (%) = CTCs captured in the chip/(CTCs captured in the chip + CTCs flowed out) × 100%

Isolation purity (%) = CTCs captured in the chip/total cells captured in the chip (including RBCs, WBCs, and CTCs) × 100%

### 2.3. Shear Stress

The core calculation formula for fluid shear stress is $\tau =\mu \frac{du}{dy}$, which means that the magnitude of shear stress depends on the viscosity of the fluid and change of the speed of velocity.

$$\tau =\mu \frac{du}{dy}$$

τ represents shear stress, which is the force generated by the friction between fluid layers, measured in pascals (Pa).

μ represents dynamic viscosity, a physical quantity that reflects the viscosity of a fluid, measured in pascals ∗ second (Pa · s).

$\frac{du}{dy}$ represents velocity gradient (also known as shear rate), describing the speed at which fluid velocity varies with distance, measured in seconds (s^−1^).

## 3. Results and Discussion

### 3.1. Design and Working Principle of Flower-Shaped Microfluidic Chip

**Figure 2:**
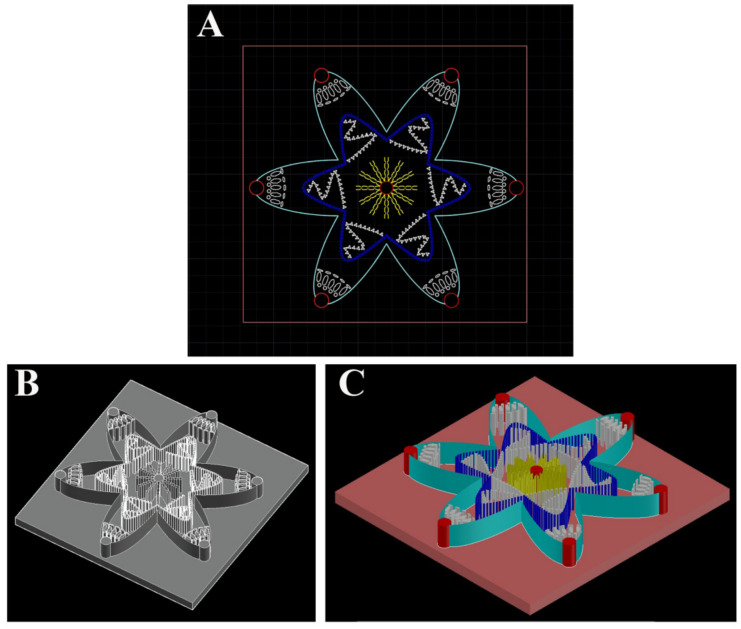
Structure of a multifunctional double-array petal flower-shaped microfluidic chip. (**A**) Overall diagram of a double-array petal flower-shaped microfluidic chip. The middle is inlet and there are six petals. (**B**) Grayscale image of the double-array multifunctional chip. (**C**) Stereogram of the multifunctional microfluidic chip.

**Figure 3:**
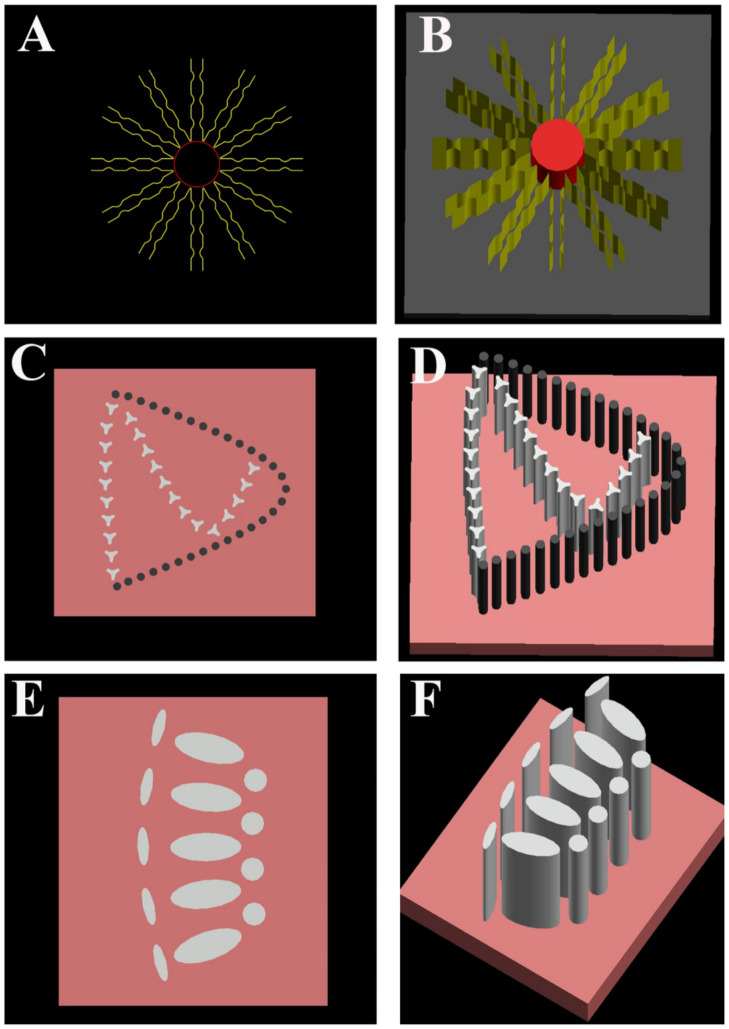
The top view and three-dimensional view of the multifunctional chip. The top view of (**A**) the first structure, the radioactive part (**C**) the second structure, S-shaped concave triangle microposts (**E**) third structure of elliptical and circular microposts, correspondingly. The three-dimensional view of (**B**) the first structure, (**D**) the second structure, and (**F**) the third structure, correspondingly.

**Figure 4:**
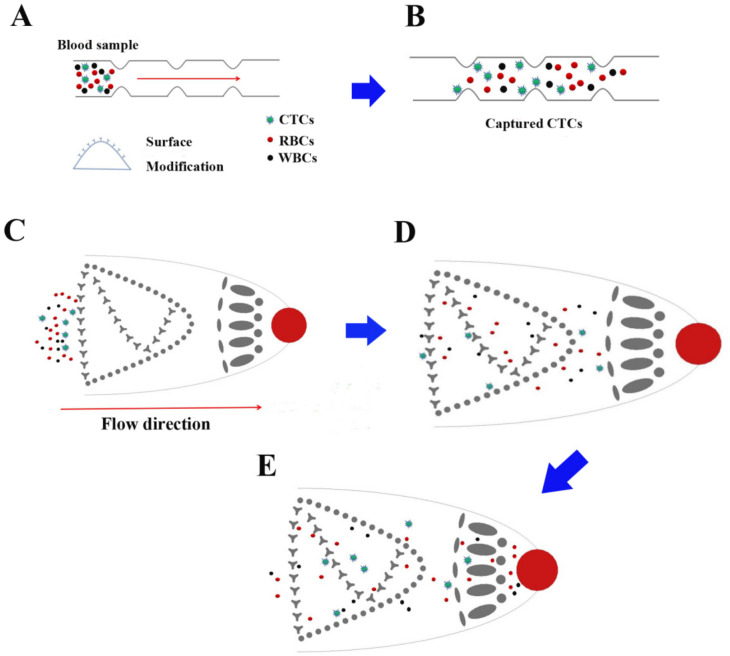
Working principle of the multifunctional double-array petal flower-shaped microfluidic chip (**A**) whole blood samples enter one of the modified radioactive microchannels. (**B**) CTCs interact with anti-EpCAM modified protuberant part and the microchannel, and CTCs are captured. (**C**) Whole blood samples flow to the front of the S-shaped petal-shaped structures. (**D**) CTCs are captured by the S-shaped array composed of concave triangle microposts. (**E**) CTCs are captured by the arrays of elliptical microposts and blood cells are depleted.

The novel flower-shaped microfluidic chip is composed of three structures of different arrays, as shown in Figure 2 and Figure 3. The first capture structure is based on the principle of affinity. Six convex structures are set in each narrow channel. The spacing of the opposite convex structures is 8 μm and the height is 100 μm. The first structure has a total of 12 such channels, which can increase collision frequency between cancer cells and convex structures in the channel. On the surface of each convex structure, the antibody corresponding to the antigen on the surface of CTCs is modified. The antibody specifically binds with the antigen, and the circulating tumor cells can be captured to a large extent, as shown in Figure 4. After the first capture, the blood is divided into 12 branches and flows to next capture structure to improve the capture rate.

The second petal capture structure is a structure of an S-shaped array composed of concave triangular microposts and parabolic array of circular microposts. In this structure, a total of six are arranged in the chip to form the first array of petals inside. The length of the curved shaped array of the concave triangular micropost is 2 mm, the height is 100 μm, the diameter of the cylinder micropost is 50 μm, and the distance between all adjacent microposts is 5 μm. The six branches contain a large number of microposts, forming a larger capture space, which greatly improves capture efficiency.

The third capture structure is composed of elliptical microposts and cylinders. The treated blood is captured for the last time. The distance between adjacent microposts is 5 μm. The long axis length of the large elliptical micropost is 40 μm, the short axis length is 20 μm, the long axis length of the small elliptical micropost is 20 μm, and the short axis length is 10 μm. The cylinder diameter is 50 μm and the height of all microposts is 60 μm. Because of the round shape, it can retain cell viability to a great extent, which is convenient for the later pathological analysis of tumor cells.

The working principle of this multifunctional double-array petal flower-shaped microfluidic chip is described as shown in Figure 4. It combines both physical and affinity properties of CTC isolation. When patient blood samples are introduced from the center inlet, antigens of CTCs such as EpCAM (Epithelial C Epithelial Cell Adhesion Molecule) are bonded with antibody-modified channel surfaces such as anti-EpCAM. CTCs are blocked by and have a collision with the protuberant parts, and then slow down and recline on the small hills. WBCs (white blood cells) and RBCs (red blood cells) flow freely away from the central line.

The second structure is S-shaped concave microposts enclosed by a parabolic circular microposts array. CTCs could be captured by grooves of concave triangle microposts, or the gaps of 5 μm formed by adjacent microposts. Relatively small sized and more deformable WBCs and RBCs could pass away. The parabolic circular array segregates CTCs once again.

For the third structure, ellipse, inverted ellipse and circular microposts are organized close to the outlet. Big elliptical microposts are utilized to function as flow diversion. Therefore, the diverting flow has to transverse out from the gaps formed by neighboring microposts. Blood cells flow easily away from the outlet.

### 3.2. Simulation of Flower-Shaped Microfluidic Chip

Through COMSOL simulation, pressure field, velocity field and streamline behaviors and shear influence for the flower-shaped microfluidic chips could be accomplished. Software parameters are set at 1054 kg/m^3^ for liquid density, 4 × 10^−3^ Pa · s for dynamic viscosity, and 2.778 × 10^−10^ m^3^/s for the velocity of the inlet.

#### 3.2.1. Numerical Analysis of the First Structure

**Figure 5:**
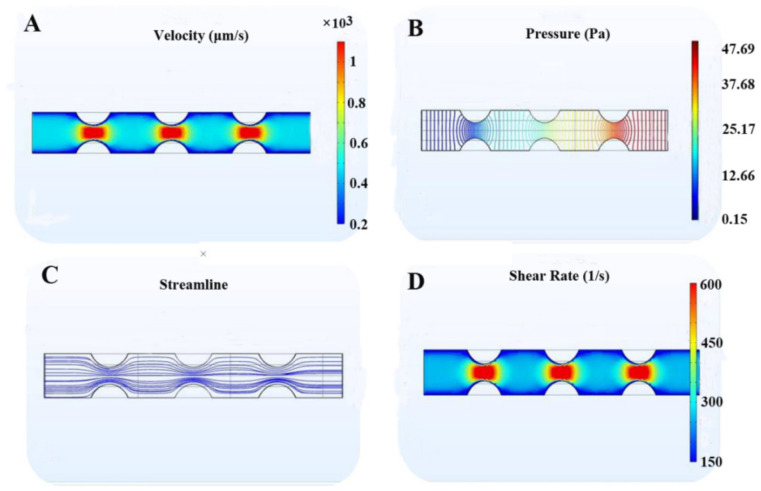
Simulation of the first structure composed of 12 radioactive microchannels with prominent posts. (**A**) Velocity behavior (μm/s), (**B**) pressure effect (Pa), (**C**) streamline tendency, (**D**) shear rate (1/s).

From the color distribution in the flow velocity simulation diagram in Figure 5A, it can be seen that in the whole channel the six bulge structures show a comprehensive color of red, orange and yellow. Other places in the channel generally show light blue and dark blue. According to the speed distribution proportion bar, the flow velocity at the bulge is large. The flow velocity is 0.8 × 10^3^ μm/s~1.1 × 10^3^ μm/s, and the flow rate in other places is 0.3 × 10^3^ μm/s~0.5 ×10^3^ μm/s. The blood flow rate in the channel is relatively slow, which ensures that the blood has sufficient contact time with the convex structure. Because the tumor cells captured at the convex are based on the principle of antigen–antibody binding, the longer the contact time is, the higher the capture rate will be, and these two are in direct proportion. In addition, the flow velocity of the three bulges is faster than that of other parts in the channel, indicating that the blood flow of the whole channel is relatively smooth, so after the tumor cells are captured, the remaining uncapped tumor cells, as well as red blood cells (RBCs) and white blood cells (WBCs) in the blood, can smoothly enter next capture structure.

From the color distribution in the pressure simulation diagram in Figure 5B, we can see that the color of the pressure curve in the whole channel is roughly from blue to light blue to yellow and then to red from left to right. According to the pressure distribution proportion bar, we can know that the pressure in the channel is increasing from left to right, with a range of approximately 2.65 Pa~45.18 Pa. When the blood just enters the channel from the inlet, due to the sample passing a small distance, the initial pressure is small, which leads to a slow flow rate. Because the front convex structure is based on the affinity principle to capture tumor cells, the low initial pressure and slow flow rate increase the specific binding time of tumor cell antigen–antibody and improve capture efficiency. As the blood moves forward, it can be seen from the figure that the pressure is getting higher and higher, which leads to increase of blood flow rate. This ensures that un-captured CTCs and blood cells can flow out at a faster flow rate and flow to the next structure. Capture efficiency will increase, and the overall operation process is reasonable.

From the streamline distribution in the streamline simulation diagram in Figure 5C, it can be seen that the distribution of the convex outflow streamline is relatively crowded, indicating that the tumor cells, RBCs and WBCs are also crowded when they reach the bulge. Once crowded, collision frequency between tumor cells and the bulge will be greatly increased. Due to the affinity principle, the capture rate of tumor cells by bulge will be significantly improved.

From the color distribution of the shear force simulation diagram in Figure 5D, it can be seen that the color distribution is roughly the same as that of flow velocity diagram. The channel is generally light blue, and the bulge is a comprehensive color of green, yellow and red. From the shear force scale bar, it can be seen that the shear rate in the channel is between 200/s and 300/s, and the bulge shear rate is between 400/s and 600/s. The shear force represents the viscous resistance of blood. The first structure uses the principle of antibody and antigen binding at the bulge to capture. The greater the shear force, the longer the tumor cells, RBCs and WBCs stay when flowing forward through the bulge due to inertia, which increases time for antigen–antibody binding, thereby improving capture efficiency.

#### 3.2.2. Numerical Analysis of the Second Structure

**Figure 6:**
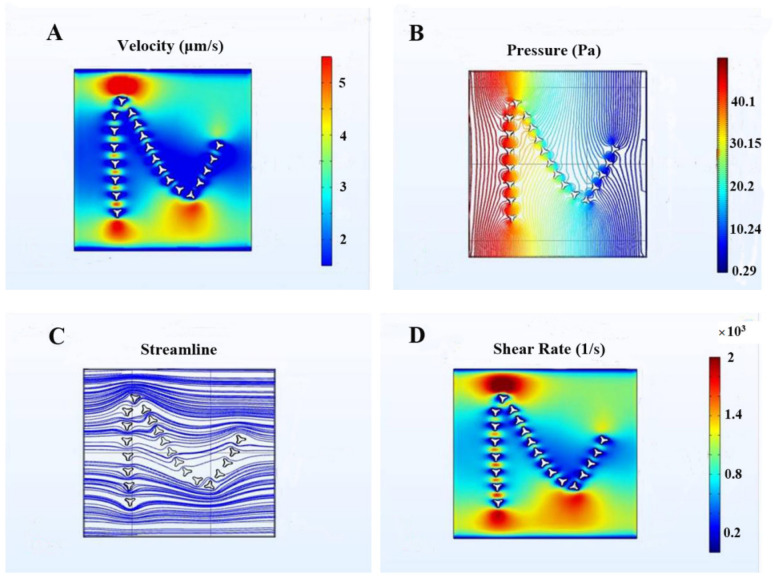
Simulation of the second structure of S-shaped array organized by concave triangle microposts. (**A**) Velocity (μm/s), (**B**) pressure (Pa), (**C**) streamline, (**D**) shear rate (1/s).

From the color distribution in Figure 6A, it can be seen that the place where the blood passes is roughly blue, and the color is red and yellow when it reaches the space between the curved triangular microposts initially. The flow rate in the blue position is slow, and the flow rate range is between 6 μm/s and 10 μm/s. The flow rate in the red and yellow positions is fast, and the flow rate range is between 16 μm/s and 20 μm/s. In other words, when the blood flowing from the first structure enters the second one, it enters at a slow speed. When passing through the first gap, the flow rate increases sharply, and the tumor cells, RBCs and WBCs in the blood will quickly pass through the first gap. Because the distance between the curved triangular microposts is set at 5 μm, some tumor cells will be stuck by the first gap and other cells will continue to flow forward to be captured by the second gap. According to the figure, the color of the second gap becomes lighter, which means that the blood flow rate becomes slower. After the flow rate becomes slower, CTCs are easier to be captured when passing through the gap, and so on. More and more CTCs will be captured in the third gap, and the capture ability will increase.

From the pressure simulation diagram in Figure 6B, we can know the color distribution of the pressure curve. On the whole, from left to right, the color is from red to yellow, then to light blue, and finally to blue. The pressure decreases from left to right as a whole. From the pressure distribution, we can know that the initial pressure of blood passing through the whole structure is large. Under the action of large pressure, tumor cells, RBCs and WBCs quickly pass through the first gap, and some CTCs are captured by the first gap. Due to the high pressure, tumor cells will also be easy to be pressed away, but the remaining cells flow into the following three gaps, as the pressure of blood flowing to the right becomes smaller and smaller, the probability of CTCs being pressed away becomes smaller and smaller; that is, the capture ability of the structure becomes larger and larger in the whole process, which ensures both the flow rate and capture efficiency. Then, from analysis of both sides of the gap, the color distribution on both sides of each gap represents that there is a pressure difference on both sides, and the pressure difference is relatively obvious. It is precise because of the pressure difference that blood cells can smoothly pass through each gap, ensuring the smoothness of the overall flow. To sum up, the overall capture rate and efficiency of the second S-shaped array of concave triangular microposts are relatively good, which has certain advantages.

From the streamline distribution in the streamline simulation diagram in Figure 6C, it can be seen that the streamline distribution between the posts is relatively dense, indicating that cells are easy to get stuck when passing through the gap, and the remaining cells flow forward and continue to be captured by next gap. The streamline diagram shows the rationality of this structure capture.

From the color distribution of the shear stress simulation diagram in Figure 6D, it can be seen that the color distribution is similar to that of the flow velocity diagram. The channel is generally light blue, and the gap is a comprehensive color of yellow and red. From the shear force proportional bar, it can be seen that the shear rate at the channel is approximately 0.2 × 10^3^/s~0.4 × 10^3^/s and the shear rate at the first gap is approximately 1.2 × 10^3^/s~1.4 ×10^3^/s. As explained above, the shear force represents the viscous resistance of blood at the gap. The greater of the resistance, the greater of the friction of blood flow. At the first gap, due to the higher pressure, the friction between cells and the gap increases. This makes it difficult for cells to flow away and easier for them to be captured by the gap. As the blood flows to the right, the shear force at the gap decreases, the resistance of cells decreases, and the flow rate increases. The combination of the two improves the capture efficiency and flow rate of the structure. The overall structure is efficient and reliable.

#### 3.2.3. Numerical Analysis of Petal-Shaped Array Composed of Circular Microposts

**Figure 7:**
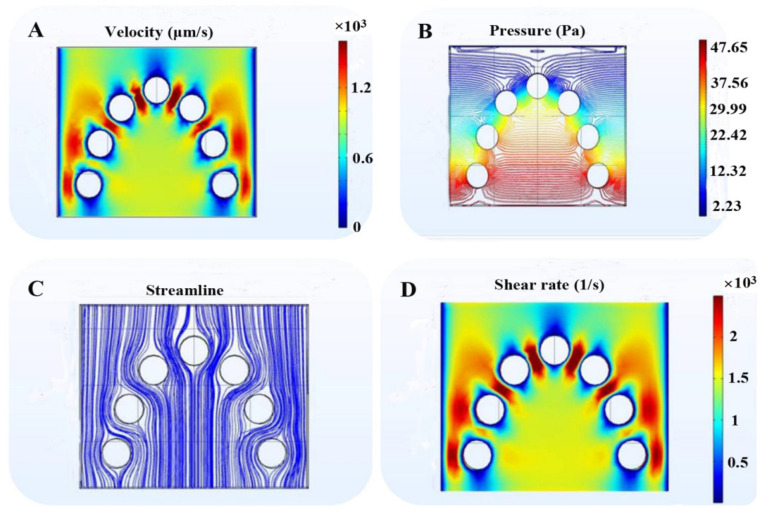
Simulation of the second structure of half circular array composed of circular microposts. (**A**) Velocity behavior (μm/s), (**B**) pressure effect (Pa), (**C**) streamline tendency, (**D**) shear rate (1/s).

From the velocity simulation diagram in Figure 7A, it can be seen that the color at the cylinder gap is darker than that at other places in the channel, indicating that velocity at the gap is relatively larger. The function of the cylindrical microposts is to capture CTCs in the blood captured by the S-shaped array of concave triangular microposts. It can be seen from color distribution that the blood passing through first structure flows to the second structure at a faster speed, but the color is darker at the gap, and speed becomes larger relatively. In this way, a small number of remaining tumor cells will be stuck by the gap formed by cylindrical posts. At the same time, RBCs and WBCs will flow to the next structure at a relatively high speed.

From the pressure simulation diagram in Figure 7B, it can be seen that on the whole, the color from bottom to top is from red to yellow, then to light blue, and finally to blue, and the pressure decreases from bottom to top. From pressure distribution, it can be seen that when the blood passes through, the inner side of the array of cylinders is red as a whole, and the pressure is large, roughly distributed between 32.51 Pa and 47.65 Pa, and the outer side of the array of cylinders is blue, roughly distributed between 2.23 Pa and 22.42 Pa. Due to the large internal and external pressure difference, it makes the blood flow from faster to slower. This would be beneficial for the tumor cells to be stuck when flowing through the gap. Once captured, it is difficult for CTCs to be missed, and the blood can flow to the last structure at a faster speed.

From the streamline distribution in the streamline simulation diagram in Figure 7C, it can be seen that the overall streamline is evenly distributed, indicating that when the blood just enters this region, it flows in a relatively parallel state, and the blood flow state is relatively average. The advantage of this is that when the blood cells enter the third structure, they are in average contact with the third structure, which is conducive to the capture of tumor cells and improves the capture rate. From this figure, it also can be seen that the streamline distribution in the circular micropost gaps is relatively dense, indicating that tumor cells are easily captured by the gaps. CTCs have no choice except passing through the gaps to be seized and blood cells flow away.

From the shear force simulation diagram in 7D, it can be seen that the color of the gap formed by circular microposts is the darkest, indicating that the shear force is the largest. As explained above, the shear force represents the viscous resistance received by the blood sample in the gap. The greater the resistance, the greater the friction force received by the blood flow. The increase of friction force makes it difficult for cells to flow away and easier for them to be captured by the circular micropost gap, and improves the capture rate.

#### 3.2.4. Numerical Analysis of the Third Array

**Figure 8:**
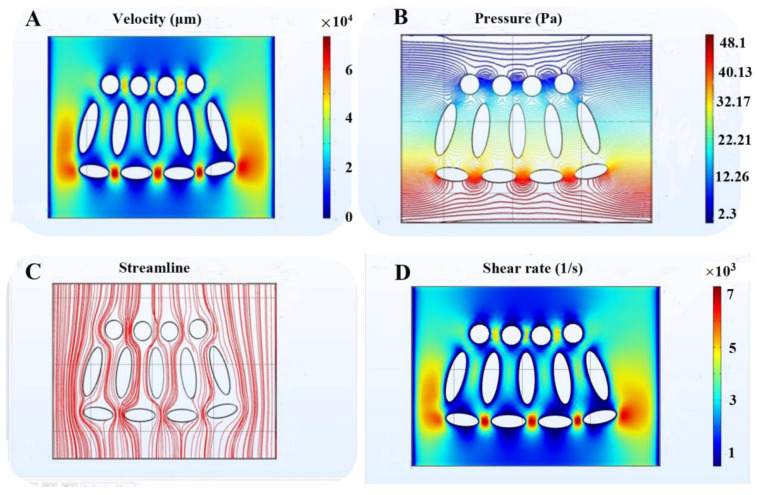
Simulation of the third structure of three arrays composed of elliptical and circular microposts. (**A**) Velocity field (μm/s), (**B**) pressure field (Pa), (**C**) streamline tendency, (**D**) shear rate (1/s).

From the velocity simulation diagram in Figure 8A, it can be seen that the color distribution of blood before reaching the elliptical micropost is roughly blue, and the flow rate is slow. When the blood flows to the first row of elliptical microposts, the gap flow rate reaches the maximum, and the gap flow rate of the second row and the third row of microposts decreases. The high flow rate of the first row ensures the capture rate of tumor cells by the microstructure, and the decreasing flow rate ensures that the latter structure can capture tumor cells to the greatest extent, so as to achieve efficient capture.

From the pressure simulation diagram in Figure 8B, it can be seen that the pressure decreases as a whole from bottom to top. From the pressure distribution, it can be seen that when the blood passes through, there is a large pressure difference. This indicates that when the tumor cells tend to be captured flowing through the micropost gap, RBCs and WBCs can flow to the outlet at a faster speed to complete the whole capture process.

From the streamline simulation diagram in Figure 8C, it can be seen that streamline shuttles in the gap area between microposts. When passing through the whole gap, a bunch of streamline has three dense places, which can repeatedly capture tumor cells, and the capture rate is high.

From the shear force simulation diagram in Figure 8D, it can be seen that the color at the gap of the elliptical micropost is the darkest, indicating that the shear force is the largest; that is, the resistance is greatest. From the overall color, the shear force decreases. At the beginning, the resistance to blood flow is greater, and the increased resistance makes it difficult for cells to flow away and easier to be captured by the gap of the elliptical microposts. Then the resistance decreases, and the flow rate increases, which improves the capture rate.

## 4. Discussion

In this work, we proposed a double-array petal flower-shaped microfluidic chip combining both affinity and physical properties. COMSOL was is utilized to perform simulation to theoretical validation. Simulation and analysis of velocity field, pressure field, streamline tendency and shear rate were carried out. From theoretical analysis, the capture principle, capture rate and capture efficiency of the chip could be comprehended. For the first structure of the design, there are 12 microchannels arranged in radiance, which could improve the flow rate. Prominent design would facilitate antigen–antibody binding. According to the simulation results, it can be shown that this structure has strong capture ability. The advantage of the first structure is the radiance of 12 channels, which improve the throughput. However, the specific structure could be redesigned to be slightly complicated to enhance contact chances between CTCs and the surfaces of structures inside the channel. The subsequent structures are based on the physical size to capture tumor cells. The second one is composed of an S-shaped micropost array of concave triangles. They are enclosed by a parabolic circular micropost array. The third structure mainly contains elliptical and inverted elliptical microposts, which make diversion and segregation easier. Due to the petal structure, the number of microposts is large, which can realize numerous captures. The design of the second structure is ingenious. The S-shaped concave micropost array is circuitous, and surrounded by the parabolic circular microposts array. The gap distance could be set at 8 μm for neighboring microposts. The improvement would be to alter the parabolic circular microposts into other shaped microposts to strengthen capture of missed CTCs. The third structure is well designed to realize both size-based capture and high flow rate. However, the ability of circular microposts to block CTCs is limited because CTCs are easy to extrude out the circular microposts, thus weaken to seize missed CTCs. It would be improved if pea-shaped microposts or cross-shaped microposts were used. Both the microposts and the shape of the design are regular. Therefore, there is no potential experimental challenge associated with the design. Eventually, those three component structures could be reasonably optimized according to the results shown in the simulation diagram. Based on the above results, the chip design is reasonable and feasible, ready for the following fabrication and clinical enumeration.

## 5. Conclusions

This design is a double-array petal flower-shaped microfluidic chip based on affinity and physical properties. Based on the physical size, the appropriate spacing is set according to the size of CTCs and various blood cells, and the corresponding antibody of tumor cells is modified on the protruding surface of 12 microchannels. The antigen on the surface of tumor cells is specifically binding with the antibody, coupled with the capture of tumor cells by physical properties. The incoming blood is screened for many times to achieve high-speed and high-efficiency capture. The advantage of the chip structure is that the affinity and physical size methods are used to screen circulating tumor cells on one plane at the same time. At the same time, the microposts of the middle level are composed of two layers of nested microposts to realize multiple captures. Finally, the structure composed of elliptical microposts and cylinders is used for the third capture. Considering the viability of tumor cells after capture, all microposts are designed with round edges. This aims to improve the capture rate while maintaining cell viability. Experimental validation using actual CTCs is feasible and essential for evaluating capture efficiency. Manufacturability and experimental data would be included when experimental conditions are available.

## Data Availability

The data that support the findings of this study are available from the corresponding author upon reasonable request.

## References

[1] Plaks V., Koopman C.D., Werb Z. (2013). Circulating tumor cells. Science.

[2] Conteduca V., Zamarchi R., Rossi E., Condelli V., Troiani L., Aieta M. (2013). Circulating tumor cells: Utopia or reality?. Future Oncol..

[3] Pantel K., Brakenhoff R.H., Brandt B. (2008). Clinical relevance and specific biological properties of disseminating tumour cells. Nat. Rev. Cancer.

[4] Mehlen P., Puisieux A. (2006). Metastasis: A question of life or death. Nat. Rev. Cancer.

[5] Barradas A.M.C., Terstappen L.W.M.M. (2013). Towards the biological understanding of CTC: Capture technologies, definitions and potential to create metastasis. Cancers.

[6] Joyce J.A., Pollard J.W. (2009). Microenvironmental regulation of metastasis. Nat. Rev. Cancer.

[7] Stott S.L., Lee R.J., Nagrath S., Yu M., Miyamoto D.T., Ulkus L., Inserra E.J., Ulman M., Springer S., Nakamura Z. (2010). Isolation and characterization of circulating tumor cells from patients with localized and metastatic prostate cancer. Sci. Transl. Med..

[8] Sollier E., Go D.E., Che J., Gossett D.R., O’Byrne S., Weaver W.M., Kummer N., Rettig M., Goldman J., Nickols N. (2014). Size-selective collection of circulating tumor cells using Vorex technology. Lab Chip.

[9] Qu L., Xu J., Tan X., Liu Z., Xu L., Peng R. (2014). Dual-Aptamer Modification Generates a Unique Interface for Highly Sensitive and Specific Electrochemical Detection of Tumor Cells. ACS Appl. Mater. Interfaces.

[10] Zhang Y., Li J., Cao L., Xu W., Yin Z. (2012). Circulating Tumor Cells in Hepatocellular Carcinoma: Detection Techniques, Clinical Implications, and Future Perspectives. Semin. Oncol..

[11] Wu L.J., Pan Y.D., Pei X.-Y., Chen H., Nguyen S., Kashyap A., Liu J., Wu J. (2012). Capturing Circulating Tumor Cells of Hepatocellular Carcinoma. Cancer Lett..

[12] Xu W., Cao L., Chen L., Li J., Zhang X.-F., Qian H.-H., Kang X.-Y., Zhang Y., Liao J., Shi L.-H. (2011). Isolation of Circulating Tumor Cells in Patients with Hepatocellular Carcinoma Using a Novel Cell Separation Strategy. Clin. Cancer Res..

[13] Nellore B.P.V., Kanchanapally R., Pramanik A., Sinha S.S., Chavva S.R., Hanne A., Ray P.C. (2015). Aptamer-Conjugated Graphene Oxide Membranes for Highly Efficient Capture and Accurate Identification of Multiple Types of Circulating Tumor Cells. Bioconjugate Chem..

[14] Habli Z., AlChamaa W., Saab R., Kadara H., Khraiche M. (2020). Circulating Tumore Cell Detection Technologies and Clinical Utility: Challenges and Opportunities. Cancer.

[15] Cristofanilli M., Budd G.T., Ellis M.J., Stopeck A., Matera J., Miller M.C., Reuben J.M., Doyle G.V., Allard W.J., Terstappen L.W.M.M. (2004). Circulating tumor cells, disease progression, and survival in metastatic breast cancer. Engl. J. Med..

[16] Khoja L., Lorigan P., Zhou C., Lancashire M., Booth J., Cummings J., Califano R., Clack G., Hughes A., Dive C. (2013). Biomarker utility of circulating tumor cells in metastatic cutaneous melanoma. J. Investig. Dermatol..

[17] de Wit S., van Dalum G., Lenferink A.T.M., Tibbe A.G., Hiltermann T.J.N., Groen H.J.M., van Rijn C.J.M., Terstappen L.W.M.M. (2015). The detection of EpCAM(+) and EpCAM(−) circulating tumor cells. Sci. Rep..

[18] Cohen S.J., Punt C.J.A., Lannotti N., Saidman B.H., Sabbath K.D., Gabrail N.Y., Picus J., Morse M.A., Mitchell M.E., Miller M.C. (2009). Prognostic significance of circulating tumor cells in patients with metastatic colorectal cancer. Ann. Oncol..

[19] Murlidhar V., Zeinali M., Grabauskiene S., Ghannad-Rezaie M., Wicha M.S., Simeone D.M., Ramnath N., Reddy R.M., Nagrath S. (2014). Radial flow microfluidic device for high-throughput affinity-based isolation of circulating tumor cells. Small.

[20] Tan K., Leong S.M., Kee Z., Caramat P.V., Teo J., Blanco M.V.M., Koay E.S.C., Cheong W.K., Soh T.I., Yong W.P. (2018). Radial flow microfluidic device for high-throughput affinity-based isolation of circulating tumor cells. Cancer Lett..

[21] Stott S.L., Hsu C.-H., Tsukrov D.I., Yu M., Miyamoto D.T., Waltman B.A., Rothenberg S.M., Shah A.M., Smas M.E., Korir G.K. (2010). Isolation of circulating tumor cells using a microvortex-generating herringbone-chip. Proc. Natl. Acad. Sci. USA.

[22] Yoo H.J., Kim T.H., Zhang Z., Azizi E., Nagrath S. (2013). Sensitive capture of circulating tumor cells by functionalized grapheme oxide nanosheets. Nat. Nanotechnol..

[23] Zheng F., Cheng Y., Wang J., Lu J., Zhang B., Zhao Y., Gu Z. (2014). Aptamer-functionalized barcode particles for the capture and detection of multiple types of circulating tumor cells. Adv. Mater..

[24] Nagrath S., Sequist L.V., Maheswaran S., Bell D.W., Irimia D., Ulkus L., Smith M.R., Kwak E.L.K., Digumarthy S., Muzikansky A. (2007). Isolation of rare circulating tumor cells in cancer patients by microchip. Nature.

[25] Ishibashi R., Yoshida S., Odawara N., Kishikawa T., Kondo R., Nakada A., Hakuta R., Takahara N., Tanaka E., Sekiba K. (2019). Detection of circulating colorectal cancer cells by a custom microfluid system before and after endoscopic metallic stent placement. Oncol. Lett..

[26] Chen K., Dopico P., Varillas J., Zhang J., George T.J., Fan Z.H. (2019). Integration of lateral filter arrays with immunoaffinity for circulating-tumor-cell isolation. Angew. Chem. Int. Ed. Engl..

[27] Sarioglu A.F., Aceto N., Kojic N., Donaldson M.C., Zeinali M., Hamza B., Engstrom A., Zhu H., Sundaresan T.K., Miyamoto D.T. (2015). A microfluidic device for label-free, physical capture of circulating tumor cell clusters. Nat. Methods.

[28] Lim L.S., Hu M., Huang M.C., Cheong W.C., Gan A.T.L., Looi X.L., Leong S.M., Koay E.S.-C., Li M.-H. (2012). Microsieve lab-chip device for rapid enumeration and fluorescence in situ hybridization of circulating tumor cells. Lab Chip.

[29] McFaul S.F., Lin B.K., Ma H.S. (2012). Cell separation based on size and deformability using microfluidic funnel ratchets. Lab Chip.

[30] Zheng S.Y., Lin H.K., Lu B., Williams A., Datar R., Cote R.J., Tai Y.-C. (2011). 3Dmicrofilter device for viable circulating tumor cell (CTC) enrichment from blood. Biomed. Microdevices.

[31] Lin H.K., Zheng S., Williams A.J., Balic M., Groshen S., Scher H.I., Fleisher M., Stadler W., Datar R.H., Tai Y.-C. (2010). Portable filter-based microdevice for detection and characterization of circulating tumor cells. Clin. Cancer Res..

[32] Preira P., Grandné V., Forel J.-M., Gabriele S., Camara M., Theodoly O. (2013). Passive circulating cell sorting by deformability using a microfluidic gradual filter. Lab Chip.

[33] Magbanua M.J.M., Pugia M., Lee J.S., Jabon M., Wang V., Gubens M., Marfurt K., Pence J., Sidhu H., Uzgiris A. (2015). A novel strategy for detection and enumeration of circulating rare cell populations in metastatic cancer patients using automated microfluidic filtration and multiplex immunoassay. PLoS ONE.

[34] Lv P., Tang Z., Liang X., Guo M., Han R.P.S. (2013). Spatially gradated segregation and recovery of circulating tumor cells from peripheral blood of cancer patients. Biomicrofluidics.

[35] Tan S.J., Yobas L., Lee G.Y.H., Ong C.N., Lim C.T. (2009). Microdevice for the isolation and enumeration of cancer cells from blood. Biomed. Microdevices.

[36] Sun N., Li X., Wang Z., Li Y., Pei R. (2018). High-purity capture of CTCs based on micro-beads enhanced isolation by size of epithelial tumor cells (ISET) method. Biosens. Bioelectron..

